# Supplementary material for: Sampling and Analysis of Low-Molecular-Weight Volatile Metabolites in Cellular Headspace and Mouse Breath
Source: Metabolites. 2022 Jun 27;12(7):599. doi: 10.3390/metabo12070599 (PMC9315489; doi:10.3390/metabo12070599)
Supplement: Supplementary file 1 [file metabolites-12-00599-s001.zip › metabolites-1727746-supplementary.pdf]

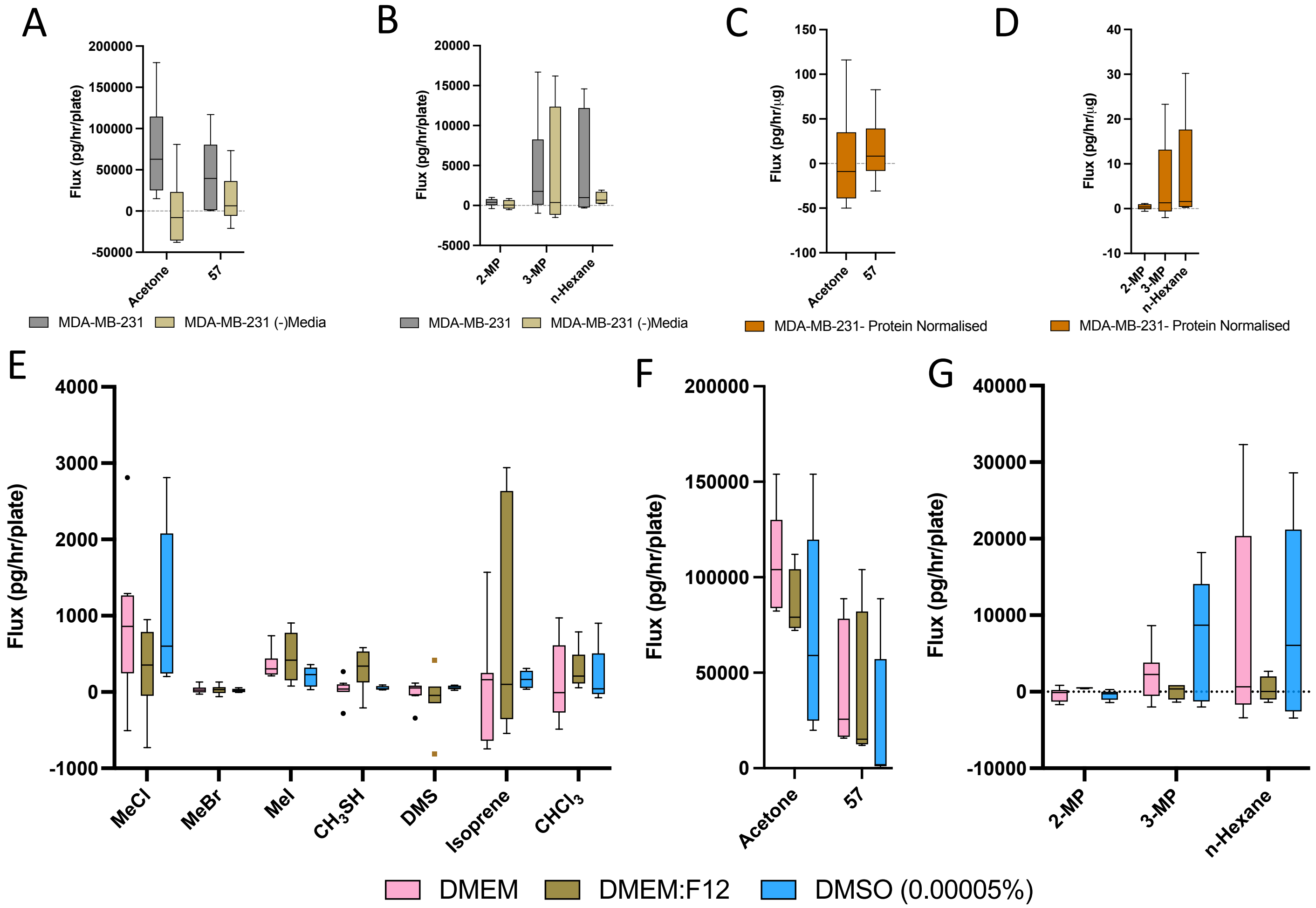

Supplementary Figure S1. Cellular volatiles and media backgrounds. Volatiles from cellular headspace vs cellular headspace with media control deducted **(A, B).** Media subtracted and protein normalised VOC flux for MCF10a (n = 9); MCF7 (n = 4); MDA-MB-231 cells (n = 6) **(C,D)**. Volatiles released from media alone. DMEM (n = 6), DMEM:F12 (n = 4), DMSO addition (n = 6) **(E, F, G).** CHCI3 = Chloroform, DMS = Dimethyl sulfide, MeBr = Methyl bromide, MeCl = Methyl Chloride, MeI = methyl iodide, MeSH = Methanoethiol Boxplot whiskers show median ± Tukey distribution. ANOVA followed by Tukey or Bonferroni post hoc test was performed

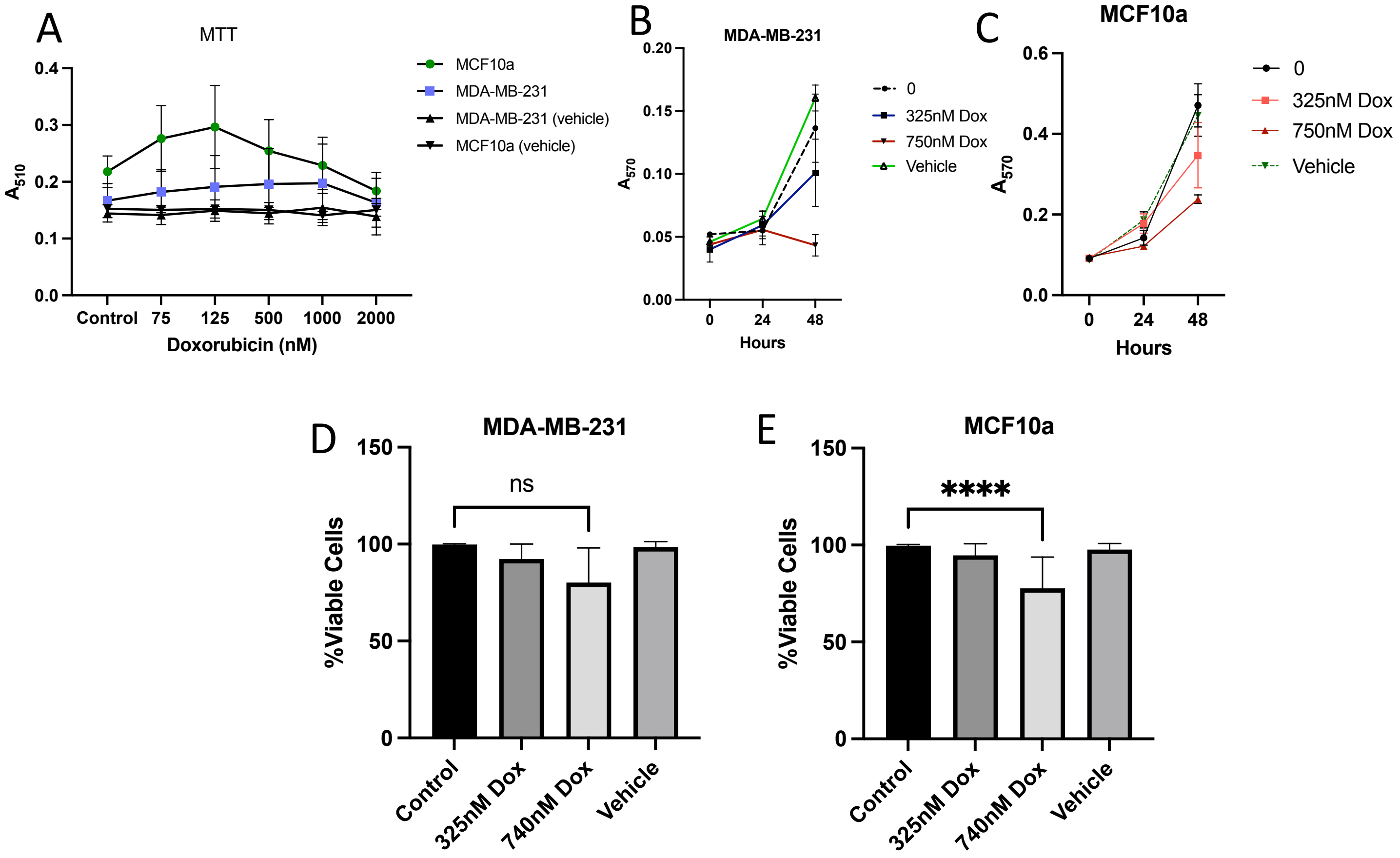

Supplementary Figure S2. Doxorubicin treatment of MDA-MB-231 and MCF10a. **(A)** MTT assay of varying concentrations of doxorubicin for both MDA-MB-231 and MCF10a. Relative levels of vehicle (DMSO) are provided at each stage (mean ± SEM; n = 3). **(B, C)** Sulforhodamine B assay over time for with doxorubicin treatment (DOX) and vehicle (DMSO, 0.00008%) for MDA-MB-231 and MCF10a cells (mean ± SEM; n = 3). **(D, E)** Trypan blue exclusion assays following 24hr doxorubicin treatment and vehicle (DMSO, 0.00008%) for MDA-MB-231 and MCf10a. ANOVA followed by Bonferroni post hoc test was performed; ****$p < 0.0001$.
